# A Broken Heart: A Case of Takotsubo Cardiomyopathy

**DOI:** 10.7759/cureus.19933

**Published:** 2021-11-27

**Authors:** Eric Landa, Erika Vigandt, Luis Moron, Manveer Ubhi, Basilides Fermin

**Affiliations:** 1 Internal Medicine, Wyckoff Heights Medical Center, New York, USA; 2 Internal Medicine, Unity Health Hospital, Searcy, USA; 3 Internal Medicine, The Brooklyn Hospital Center, New York, USA

**Keywords:** takotsubo syndrome, broken heart syndrome, stress-induced cardiomyopathy, reversible heart failure, takotsubo cardiomyopathy (ttc)

## Abstract

Emotional stress-induced left ventricular dysfunction, also known as Takotsubo cardiomyopathy, is a condition that has become more prevalent since the turn of the century. Seen most commonly in postmenopausal women who experience an acute emotional stressor, its presentation resembles that of acute coronary syndrome with electrocardiogram changes, which is why most patients receive a left heart catheterization revealing clean or nonobstructive coronary arteries.

## Introduction

Also known as a transient apical ballooning syndrome, stress-induced cardiomyopathy, and broken heart syndrome, Takotsubo cardiomyopathy is a form of nonischemic cardiomyopathy characterized by transient regional systolic dysfunction of the left ventricle in the absence of significant coronary artery disease (CAD) [1]. This causes ballooning of the apex, making the resemblance of a jar used to trap Japanese octopus, hence the name. Causing a reduction in the ejection fraction, it results in reversible heart failure that self-resolves in a matter of weeks [1,2]. In 1990, Sato et al. first described this reversible cardiomyopathy as "tako-tsubo-like" left ventricular dysfunction, outside Japan.

## Case presentation

**Figure 1:**
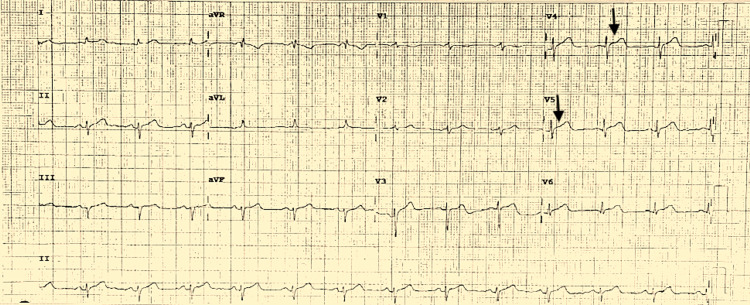
EKG demonstrating ST elevation in the lateral leads V4-V5 (arrows)

A 71-year-old female with a significant past medical history of hypertension, hyperlipidemia, and hypothyroidism presented to the emergency department with a complaint of chest pain. She had been visiting her granddaughter who was in the intensive care unit in critical condition and developed chest pain after being in significant emotional distress. A rapid response was called, and she was taken to the emergency department. The patient reported having left substernal chest pain without any radiation, 7/10 in intensity. She denied any previous history of angina or similar symptoms in the past. Her home medications included lisinopril 2.5 mg once a day, levothyroxine 88 mcg once a day, metoprolol succinate 25 mg once a day, and pravastatin 10 mg once a day. The initial vital signs were as follows: temperature, 98.1°F; blood pressure, 125/76 mmHg; heart rate, 78 beats/minute; respiratory rate, 22 breaths/minute; and oxygen saturation, 100% on room air. The laboratory values were as follow: white blood cell count, 10.2 K/uL; hemoglobin, 13.2 g/dL; sodium, 139 mmol/L; potassium, 3.9 mmol/L; BUN, 15 mg/dL; creatinine, 0.76 mg/dL; troponin, 4.0 ng/mL; and COVID-19 RT-PCR, negative. An electrocardiogram (EKG) was performed, which revealed ST elevation in the lateral leads (Figure 1).

**Figure 2:**
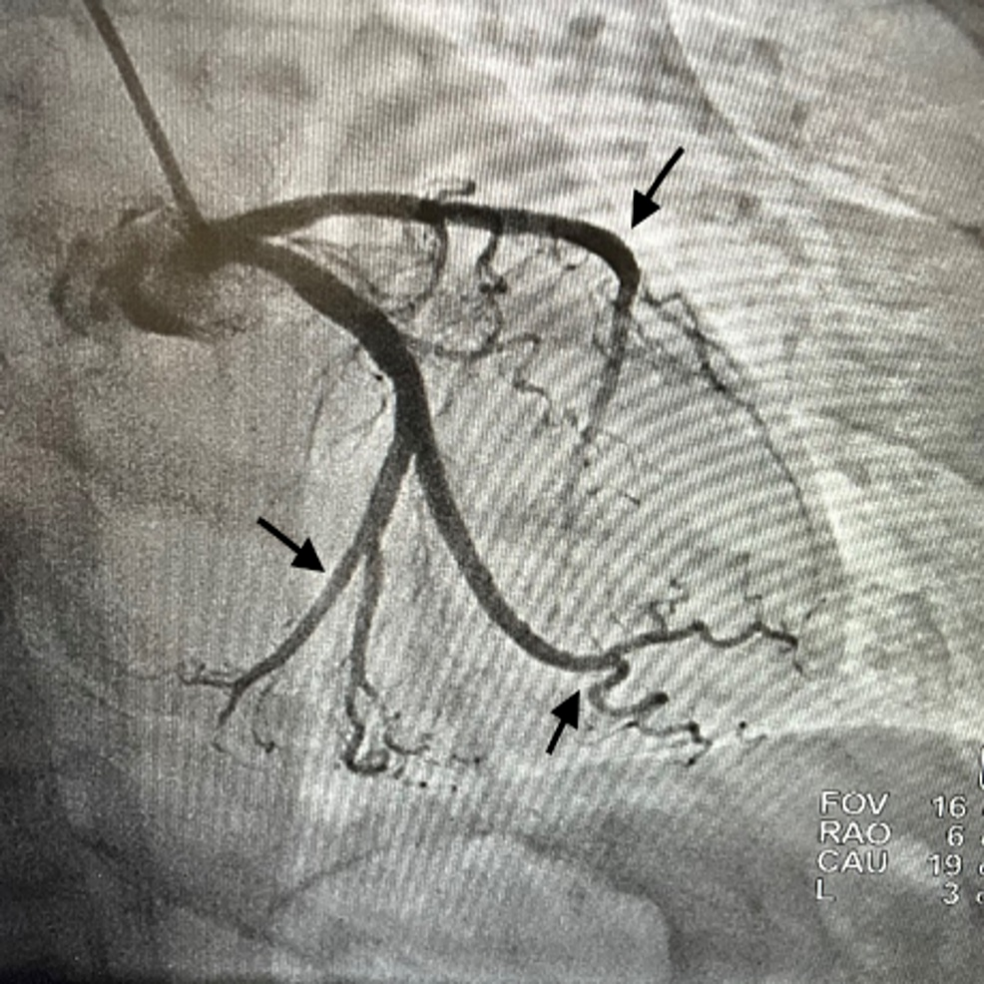
Cardiac catheterization demonstrating nonobstructive coronary artery disease (arrows)

**Figure 3:**
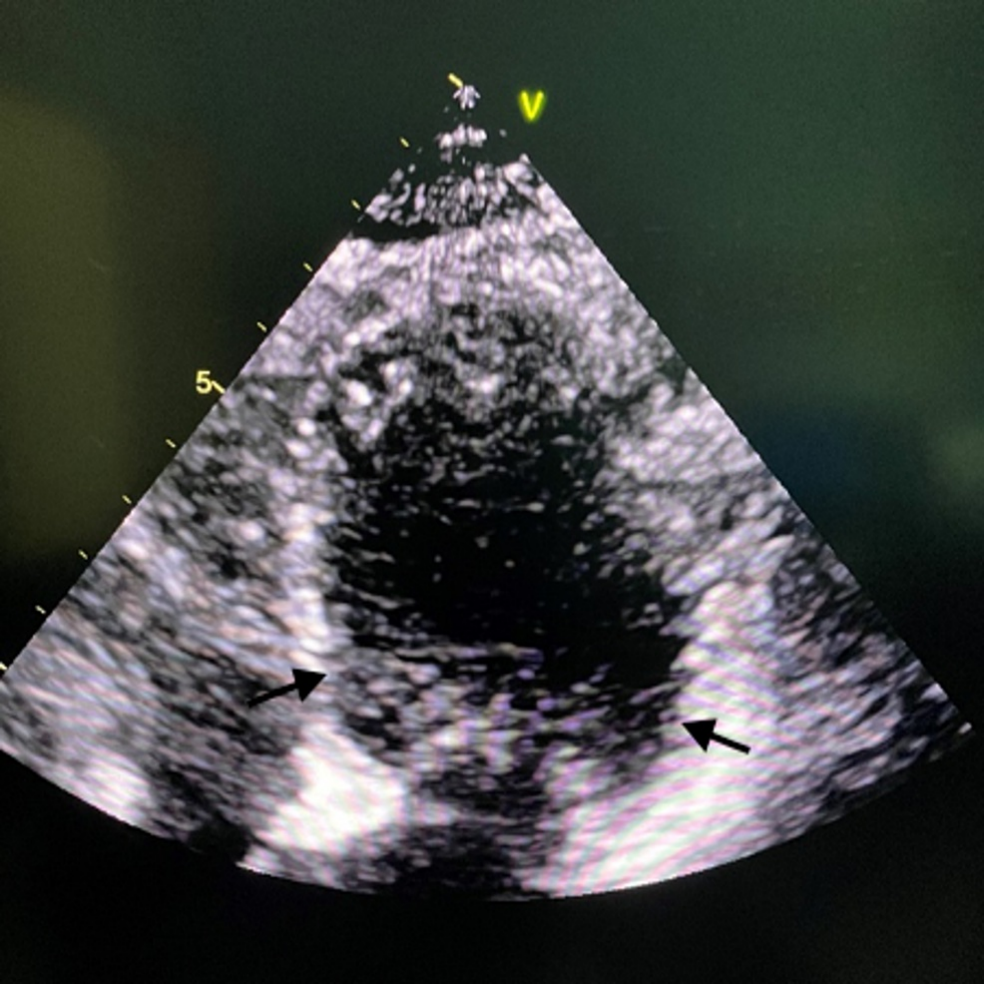
Ballooning of the left ventricle (arrow)

Given her clinical presentation along with abnormal EKG changes and elevated troponin, the patient was taken for a left heart catheterization. She was found to have Takotsubo cardiomyopathy and nonobstructive coronary artery disease (Figure 2). An echocardiogram showed an ejection fraction of 25%-30% with hypokinesis of the anterior septum, anterior lateral, and apical segment, mild regurgitation, and tricuspid regurgitation (Figure 3).

The patient was diagnosed with Takotsubo cardiomyopathy and discharged home with her home medications along with aspirin 81 mg and instructions to follow up with the cardiologist within one week.

## Discussion

The principal mechanism in Takotsubo cardiomyopathy remains unsettled. Numerous hypotheses have been proposed and include elevated levels of circulating plasma catecholamines and their circulating metabolites due to underlying stress, microvascular dysfunction, inflammation, estrogen deficiency, and spasm of the epicardial coronary vessels [1]. The catecholamine theory is the most broadly accepted pathophysiologic mechanism of Takotsubo cardiomyopathy, and elevated levels of plasma catecholamines have been seen in such patients. Estrogen applies protective effects on the cardiovascular system, including vasodilation, protection against atherosclerosis, and endothelial dysfunction [2]. Therefore, postmenopausal women display amplified vasoconstriction, altered endothelium-dependent vasodilation, and sympathetic triggering in response to psychosocial stress [3]. There currently is no set diagnostic criteria; however, researchers at the Mayo Clinic proposed a set of criteria that is commonly used (Table 1) [4].

**Table 1:** Mayo Clinic criteria for Takotsubo cardiomyopathy

Diagnostic criteria
Transient hypokinesis, akinesis, or dyskinesis in the left ventricular mid-segments with or without apical involvement; regional wall motion abnormalities that extend beyond a single epicardial vascular distribution; and frequently, but not always, a stressful trigger
Absence of obstructive coronary disease or angiographic evidence of acute plaque rupture
New EKG abnormalities (ST-segment elevation and/or T-wave inversion) or modest elevation in cardiac troponin
Absence of pheochromocytoma and myocarditis

The etiology of Takotsubo cardiomyopathy is still unsure. A systematic review of patients displaying suspected acute myocardial infarction reported a prevalence of 1.7%-2.2% [5,6]. There is a strong correlation with postmenopausal women; however, males may have a worse prognosis if affected [6]. In the International Takotsubo Registry study (a consortium of multiple centers across Europe and America of 1750 patients), approximately 88.9% of the affected patients were females, and the mean age was 66.4 years [7]. Due to its resemblance to myocardial infarction, management should center on the treatment of coronary artery disease (CAD). Thus, one of the diagnostic criteria of Takotsubo is the exclusion of CAD. For that reason, primary therapy includes oxygen supplementation, intravenous heparin, aspirin, and beta-blockers [8]. After eliminating CAD and confirming Takotsubo, aspirin can be stopped. In the case just presented, aspirin was continued as there was evidence of coronary artery disease, although it was nonobstructive. Beta-blocker usage is rational due to a high catecholamine state [9]. Additionally, angiotensin-converting enzyme inhibitors (ACE-I) and angiotensin receptor blockers (ARB) could also be used as part of regional wall motion abnormality management.

## Conclusions

Takotsubo cardiomyopathy is stress-induced cardiomyopathy that results in left ventricular dysfunction and mimics heart failure. It has a good prognosis as it is a reversible phenomenon that typically lasts a few weeks. During the past two decades, more and more cases have been recorded, and physicians along with researchers are beginning to better understand this phenomenon. It is important to teach patients the importance of stress reduction as it can harm our bodies and lead to a broken heart.
